# Oncolytic Viruses in Glioblastoma: Clinical Progress, Mechanistic Insights, and Future Therapeutic Directions

**DOI:** 10.3390/cancers17243948

**Published:** 2025-12-10

**Authors:** Jiayu Liu, Yuxin Wang, Shichao Su, Gang Cheng, Hulin Zhao, Junzhao Sun, Guochen Sun, Fangye Li, Rui Hui, Meijing Liu, Lin Wu, Dongdong Wu, Fan Yang, Yuanyuan Dang, Junru Hei, Yanteng Li, Zhao Gao, Bingxian Wang, Yunjuan Bai, Wenying Lv, Jianning Zhang

**Affiliations:** Senior Department of Neurosurgery, Chinese PLA General Hospital, Beijing 100853, China; liujiayu@pku.edu.cn (J.L.); wangyuxin037@163.com (Y.W.); shichao_2025@163.com (S.S.); yjscg2003@126.com (G.C.); zhaohulin_90@sohu.com (H.Z.); sunjunzhao77@aliyun.com (J.S.); sgc3130@126.com (G.S.); leefangye@126.com (F.L.); huirui2002@163.com (R.H.); lmj18600310571@163.com (M.L.); aman5050@sohu.com (L.W.); ddwu301@163.com (D.W.); yinciailian@126.com (F.Y.); dyy419@163.com (Y.D.); h18834189738@163.com (J.H.); yantenglibj@163.com (Y.L.); gaz3141592654@163.com (Z.G.); wangbx0730@163.com (B.W.); lang730@sina.com (Y.B.)

**Keywords:** glioblastoma, oncolytic virus, herpes simplex virus (oHSV), immunotherapy, clinical trials

## Abstract

High-grade gliomas—particularly glioblastoma (GBM)—remain refractory to standard-of-care surgery followed by chemoradiation, with a median overall survival of ~15 months. Oncolytic viruses (OVs), which selectively infect and lyse tumor cells while engaging antitumor immunity, offer a mechanistically distinct therapeutic modality. This review synthesizes clinical progress of OVs in GBM, with emphasis on oncolytic herpes simplex virus (oHSV) and coverage of other vectors (adenovirus, reovirus, Newcastle disease virus, vaccinia virus) across phase I–III trials, focusing on efficacy and safety. Key observations include the encouraging clinical trajectory of oHSV exemplars—T-VEC (approved for melanoma) and G47Δ (approved in Japan for recurrent GBM)—the multi-center exploration of the adenovirus DNX-2401 combined with programmed death-1 (PD-1) blockade, and the early-stage status of reovirus (pelareorep) and Newcastle disease virus programs. Emerging evidence indicates that oHSV therapy augments immune infiltration within the tumor microenvironment and alleviates immunosuppression, with synergy when combined with chemotherapy or immune checkpoint inhibitors. Persistent challenges include GBM’s inherently immunosuppressive milieu, limitations imposed by the blood–brain barrier, intrapatient viral delivery and biodistribution, and concerns about viral shedding. Future directions encompass programmable vector design, optimization of systemic delivery, biomarker-guided patient selection, and rational combination immunotherapy. Collectively, OVs represent a promising immunotherapeutic strategy in GBM; further gains will hinge on vector engineering and precision combinations to translate mechanistic promise into durable clinical benefit.

## Simple Summary

Glioblastoma (GBM) is the most aggressive primary brain tumor, with limited treatment options and a poor median survival of around 15 months. Oncolytic viruses (OVs) offer an innovative therapeutic approach by selectively infecting and destroying cancer cells while activating the immune system to fight tumors. This review aims to summarize the clinical progress of OVs in GBM, including platforms like oncolytic herpes simplex virus and adenovirus, and to explore mechanistic insights into how they remodel the tumor microenvironment. By highlighting recent trials and future directions, such as combination therapies, this work seeks to provide a comprehensive resource that could guide research and improve patient outcomes, potentially leading to more effective treatments for this devastating disease.

## 1. Introduction

Glioblastoma (GBM), the most common and aggressive primary malignancy of the central nervous system, remains refractory to standard therapy—including maximal safe resection followed by adjuvant chemoradiation—with a median survival of approximately 15 months, as highlighted in recent reviews on oncolytic virus advances [1,2]. This poor prognosis stems from GBM’s profound intratumoral heterogeneity, diffuse infiltration, rapid acquisition of therapy resistance, and a highly immunosuppressive tumor microenvironment characterized by abundant M2-like macrophages, regulatory T cells, elevated programmed death-ligand 1 (PD-L1), and immunosuppressive cytokines, which collectively limit the efficacy of conventional immunotherapies such as cytotoxic T-lymphocyte-associated protein 4 (CTLA-4)/programmed death-1 (PD-1) inhibitors. Oncolytic viruses (OVs), engineered to selectively replicate in tumor cells, induce lysis, and convert immunologically “cold” tumors into inflamed, T-cell-infiltrated lesions, offer a promising alternative, as highlighted in recent comprehensive reviews. This review provides a GBM-specific, platform-by-platform synthesis that integrates recent clinical advances (e.g., oHSV CAN-3110 and adenovirus DNX-2401 with PD-1 blockade) with preclinical mechanisms and biomarker-guided combination strategies. We aim to map viral entry receptors and engineered control nodes, summarize intracranial delivery options, and outline rational sequencing with radiotherapy, chemotherapy, and immunotherapies, thereby offering a practical translational framework to enhance the depth and durability of responses in GBM.

Oncolytic virotherapy represents an orthogonal therapeutic mechanism, wherein engineered OVs replicate selectively within tumor cells to induce lysis, releasing tumor antigens and danger signals that prime and amplify antitumor immune responses [3,4]. Among various platforms, oncolytic herpes simplex virus (oHSV) is particularly advanced; for instance, talimogene laherparepvec (Imlygic)—an oHSV encoding GM-CSF—has received U.S. FDA approval for melanoma, establishing clinical feasibility and an immune-stimulatory paradigm relevant to neuro-oncology [1,5]. Compared to other vectors, HSV-1 exhibits broad cellular tropism, potent oncolysis at low multiplicities of infection, availability of antiviral safety switches (e.g., acyclovir), and a large genome capable of accommodating therapeutic transgenes [3]. These attributes have been leveraged in multiple clinical attempts of OVs in GBM. Here, we present a structured appraisal of clinical progress and persistent barriers, with emphasis on oHSV. A comparative overview of key platforms, each with distinct engineering strategies and clinical trajectories, is provided in Table 1. Furthermore, oncolytic viruses engage in dynamic regulatory processes within the tumor microenvironment, such as modulating immune cell infiltration via interferon signaling and altering immunosuppressive pathways, which collectively contribute to reshaping GBM’s hostile landscape and will be elaborated in subsequent sections.

Unlike prior reviews that broadly catalog oncolytic virotherapy across solid tumors, this work advances a GBM-specific, platform-by-platform immuno-oncologic framework that integrates recent clinical updates (2023–2025), including CAN-3110 and DNX-2401 combined with PD-1 blockade, with mechanistic insights such as viral entry receptors, engineered control nodes (e.g., ICP34.5 or E1A-Δ24), interferon-type I (IFN-I, a key cytokine group in antiviral immunity)/cyclic GMP-AMP synthase-stimulator of interferon genes (cGAS-STING), and antigen spread. It also delineates biomarker-guided, trial-ready combinations and sequencing strategies, such as timing PD-1 blockade after initial intratumoral viral priming. Additionally, we highlight GBM-specific constraints—including the blood–brain barrier, myeloid-dominant suppression, neuroinflammation risk, and intracranial delivery geometry—that distinguish GBM from non-CNS cancers where OV experience is more extensive. This GBM-focused synthesis is intended as a practical design map for upcoming Phase I/II studies, rather than a general catalog, to address the unique challenges of this malignancy.

**Figure 1:**
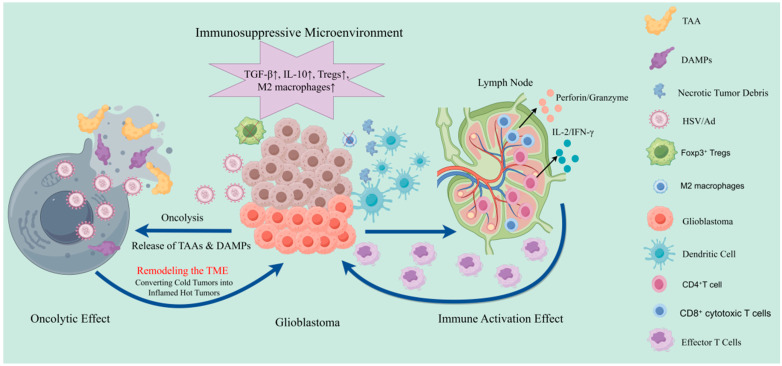
Dual antitumor mechanisms of oncolytic viruses (OVs) in glioblastoma. Arrows indicate key sequential steps or causal relationships. On the left, intratumoral viral replication directly lyses tumor cells, releasing tumor-associated antigens (TAAs) and danger-associated molecular patterns (DAMPs). On the right, these signals are captured by antigen-presenting cells (APCs), particularly dendritic cells, which activate CD4^+^ and CD8^+^ T cells. The effector lymphocytes infiltrate the tumor microenvironment, enhancing immune-mediated tumor clearance. OV therapy reshapes the immunosuppressive GBM milieu—reducing regulatory T cells (Tregs), which are immunosuppressive T cells that modulate immune tolerance, and M2 macrophages while augmenting cytotoxic T-cell infiltration—thus providing synergistic potential with immunotherapies such as PD-1 blockade.

Figure 1 aligns each OV platform with entry receptors, engineered control nodes, dominant immune axes, including IFN-I, antigen spread, and tumor-associated macrophage (TAM, immune cells that can promote tumor progression) and regulatory T cell (Treg, immunosuppressive lymphocytes) re-programming, and rational sequencing with RT and PD-1 blockade. Figure 1 illustrates the dual antitumor mechanisms of oncolytic viruses in glioblastoma, highlighting direct oncolysis and immune activation that synergize with immunotherapies such as PD-1 blockade.

## 2. Virus Platforms and Strategies

### 2.1. Oncolytic Herpes Simplex Virus (oHSV)

Oncolytic herpes simplex virus (oHSV) replication induces immunogenic tumor cell death and establishes a type-I interferon-rich microenvironment, leading to upregulation of major histocompatibility complex class I (MHC-I), promotion of CD8^+^ T-cell infiltration, and reduction in Treg and myeloid-derived suppressor cells (MDSC) burdens. Biopsy evidence following G47Δ treatment demonstrates increased CD4^+^/CD8^+^ T-cell densities with low Foxp3^+^ cell counts. CAN-3110 further associates HSV-1 seropositivity with survival benefits and shows post-dose TCR diversification, consistent with antigen-spread priming. Mechanistically, these findings support delayed PD-1 blockade (after 1–2 oHSV doses) and peri-oncolytic virotherapy low-dose radiotherapy (approximately 5 Gy) to amplify antigen release. Edema and pseudoprogression should be managed using steroid-sparing approaches, such as anti-VEGF therapy when appropriate. Intratumoral delivery methods, including multi-track injections, convection-enhanced delivery (CED), or Ommaya reservoir-facilitated repetitions, enhance coverage, while focused ultrasound-mediated blood–brain barrier opening remains exploratory.

oHSV represents one of the most extensively studied oncolytic virus platforms. Conventional constructs involve deletion or modification of loci such as γ34.5 and ICP6 to restrict replication in normal cells while preserving tumor-selective lysis [6]. Representative vectors include the triple-mutant G47Δ (featuring two γ34.5 deletions plus an ICP6/lacZ modification) and the earlier double-deleted G207. T-VEC (Imlygic), the first approved oncolytic HSV armed with GM-CSF [1], though licensed for melanoma, provides a safety and immunologic blueprint for glioblastoma (GBM) development. G47Δ has advanced clinically and received approval in Japan for recurrent or residual GBM. Next-generation oHSV candidates, such as oHSV-CN551, are under development for brain tumors. Arming strategies are central to oHSV; for example, Xu et al. engineered oHSV to express a full-length anti-CD47 IgG1, enhancing macrophage phagocytosis and NK-cell-mediated antibody-dependent cellular cytotoxicity in murine models [1], illustrating the potential of cytokine, checkpoint antibody, and other payload approaches [7].

### 2.2. Adenovirus

Conditionally replicative adenoviruses (CRAds) trigger immunogenic cell death, dendritic cell licensing, and a chemokine milieu that recruits effector T cells; however, lateral spread within the parenchyma is limited. The Δ24 E1A backbone with an RGD-modified fiber enhances integrin-mediated entry, and when combined with PD-1 blockade, survival benefits are concentrated in responders, supporting a prime-then-checkpoint sequence. CD40L or IL-12 arming can further enhance antigen presentation, while catheter reinjection/CED and mesenchymal stem cell (MSC) carriers improve distribution. Anti-adenovirus neutralizing antibodies and CAR heterogeneity underscore the need for biomarker-guided selection and timing.

CRAds achieve tumor selectivity through modifications in E1A/E1B regions. DNX-2401 (Δ24-RGD) contains an E1A deletion in the Rb-binding domain and an RGD motif to augment integrin-mediated tumor targeting [8,9]. Phase I studies have demonstrated a favorable safety profile and durable benefits in a subset of patients. Ongoing strategies include combining DNX-2401 with immune checkpoint blockade and utilizing cellular carriers (e.g., mesenchymal stem cells) to improve intratumoral distribution [10]. Additional CRAds armed with CD40L or IL-12 are under investigation.

### 2.3. Reovirus

Systemic reovirus primarily engages innate immunity and replicates preferentially in RAS-activated contexts; safety is favorable, but neutralizing antibodies and blood–brain barrier limitations constrain central nervous system efficacy. Combinations with CRT/TMZ backbones and PD-1 blockade are biologically rational, and GM-CSF may augment antigen-presenting cell recruitment. Repeated intravenous cycles, focused ultrasound-mediated BBB opening, or cell-based carriers represent delivery enhancers worthy of formal testing with immune-correlative endpoints.

As a naturally oncotropic virus, reovirus preferentially replicates in tumors with activated RAS signaling. Pelareorep (Reolysin) has been evaluated—alone or with GM-CSF or radio/chemotherapy—in GBM; to date, reports highlight safety without clear efficacy gains, necessitating larger trials [4,11].

### 2.4. Newcastle Disease Virus (NDV)

Newcastle disease virus (NDV) mediates fusogenic oncolysis and IFN-I-dominated innate activation; human GBM data remain sparse but indicate safety. Rational strategies include timed PD-1 blockade after the innate immune peak and synergy with radiotherapy; TLR3 agonists can be sequenced post-replication. Neutralization and systemic reactogenicity support dosing schedules that stagger immunotherapy and explore intranasal or focal routes.

NDV exhibits intrinsic oncolytic activity across tumor types and enhances antitumor immunity in preclinical models [12]. However, clinical experience in GBM is limited and largely preclinical, reflecting scarce human data [13,14].

### 2.5. Vaccinia Virus

Vaccinia virus, with its large genome, enables the incorporation of cytokine payloads (e.g., GM-CSF, IL-2, IL-12, IL-21) or bispecific molecules, facilitating rapid cytolysis, strong antigen presentation, and vascular remodeling. Intracranial clinical experience is limited; (brin)cidofovir serves as a pharmacologic safety backstop. Rational combinations pair vaccinia with anti-PD-1 therapy and radiotherapy; CED or intratumoral dosing mitigates neurotropism concerns and allows for theranostic imaging when NIS-like payloads are used.

Vaccinia vectors, such as Pexa-Vec in liver cancer, are attractive due to their large, engineerable genome and efficient infection, though GBM applications are nascent [15]. Armed constructs, like IL-21-expressing vaccinia combined with PD-1 blockade, show synergy in glioma models, but clinical evidence remains limited.

### 2.6. Other Vectors

PVSRIPO, a modified poliovirus targeting CD155 (often overexpressed on GBM cells), reported a 2-year overall survival of 21% in a phase I study compared to a historical rate of approximately 14% [16]. Measles virus has been assessed in various malignancies, though GBM trials have not been reported [17]. Overall, while multiple platforms are under exploration for GBM, oHSV and adenoviral vectors currently dominate the clinical landscape.

By targeting CD155 (PVR) and replacing the IRES with a rhinovirus element, PVSRIPO induces a potent IFN-I response and myeloid activation, potentially resetting the immune set-point and yielding long-tail survival plateaus in early trials. Given neutralizing immunity and procedural risks, CED with reflux minimization and delayed PD-1 blockade are rational approaches. Low-dose radiotherapy can complement antigen release, and STING/TLR agonists should be timed after the viral peak to avoid inhibiting replication.

## 3. Completed and Ongoing Clinical Studies of Oncolytic Viruses in GBM

Current clinical investigations of OVs in glioblastoma are primarily early-phase (phase I/II), with only a handful advancing to phase III or registration-level studies. Several OVs have advanced into phase I/II trials, with variable efficacy but generally favorable safety profiles. Representative trials are summarized in Table 2.

### 3.1. oHSV Clinical Trials

G207. One of the earliest oHSV constructs (double γ34.5 deletion), G207 was evaluated in Europe in a phase I trial combining intratumoral injection with focal radiotherapy. Results published in 2014 demonstrated safety without dose-limiting toxicities [28]. More recently, in 2021, a phase I pediatric study of high-risk gliomas—including diffuse intrinsic pontine glioma (DIPG)—showed that G207 was well tolerated with no DLTs, median OS ~12 months, and radiographic or clinical improvement in the majority of patients [19,20,28].

G47Δ. In a phase II single-arm Japanese trial, 19 patients with recurrent or residual GBM after radiotherapy and temozolomide received stereotactic intratumoral injections of G47Δ (up to six doses). The 1-year overall survival (OS) rate was 84.2% [29], with a median OS of 20.2 months. The most common treatment-related AEs were fever, vomiting, and leukopenia, all reversible [29]. Eighteen patients (94.7%) achieved stable disease, and one achieved partial response. On the strength of these results, G47Δ became the first OV approved in Japan for GBM [18,29].

M032. A third-generation oHSV engineered to express IL-12, currently under evaluation in pediatric solid tumors including gliomas (NCT02062827). Preliminary reports indicate good tolerability, but mature data are pending.

T-VEC. While not directly studied in large-scale GBM trials, the clinical precedent of FDA-approved T-VEC (for melanoma) [1] underscores the translational feasibility of oHSV in neuro-oncology.

CAN-3110 (linoserpaturev) is a replication-competent oHSV under the control of a nestin promoter, designed to confine viral replication to nestin-positive glioma cells. In a first-in-human phase I trial in 41 patients with recurrent GBM, intratumoral CAN-3110 was well tolerated and associated with immunoactivation; exploratory analyses linked the magnitude of intratumoral immune responses with survival outcomes. Building on these findings, serial-biopsy multiomics in a subsequent study demonstrated dynamic remodeling of the TME after CAN-3110, including increases in CD45^+^ leukocytes and interferon-response signatures, and highlighted molecular features that help distinguish pseudoprogression from true progression—information directly relevant to trial design and radiographic adjudication in GBM. Collectively, the CAN-3110 program provides clinical-grade evidence that oHSV-driven immunoactivation can be captured with paired tissue and circulating biomarkers, and supports rational sequencing with immune checkpoint blockade under iRANO-concordant assessment [21].

### 3.2. Adenovirus Clinical Trials

DNX-2401 (Δ24-RGD). In phase I studies, some patients derived long-term survival benefit. For example, in a UCLA cohort of heavily pretreated recurrent GBM, single intratumoral injections yielded survival beyond 36 months in ~20% of patients [22,23]. Building on this, DNX-2401 is being explored in multicenter phase II trials, often in combination regimens.

DNX-2401 + Pembrolizumab. A 2023 multicenter phase II trial enrolled 49 recurrent GBM patients to receive intratumoral DNX-2401 followed by intravenous pembrolizumab [23]. The combination was well tolerated, with no DLTs. The objective response rate (ORR) was 10.4% (versus a 5% benchmark), and the 12-month OS rate reached 52.7%, markedly higher than the historical ~20% expectation. Median OS was 12.5 months, with survival extension concentrated among responders (HR 0.20). Notably, three patients achieved durable remissions exceeding three years [23]. This trial highlights the potential for OV–checkpoint blockade synergy in selected patients [23,30].

Other adenoviruses. Earlier vectors such as ONYX-015 yielded limited efficacy. Ongoing studies are testing adenoviruses engineered with immune-stimulatory genes (e.g., CD40L, IL-12) or in combination with dendritic cell vaccines and chemotherapy.

### 3.3. Reovirus Clinical Trials

Pelareorep (Reolysin) has been tested in GBM, including in combination with GM-CSF and standard radio/chemotherapy. These studies confirmed acceptable safety, but efficacy gains have been modest, and no randomized evidence yet supports survival benefit [4,25].

### 3.4. Newcastle Disease Virus Clinical Trials

Clinical experience in GBM remains minimal, with only small early-phase studies reporting limited efficacy [26]. Safety appears manageable, but efficacy signals are limited, and no phase III trials have been completed to date.

### 3.5. Clinical Trials of Other Vectors

PVSRIPO (polio:rhinovirus chimera). In a phase I study, intratumoral infusion via convection-enhanced delivery yielded a 2-year survival rate of 21%—notably higher than historical 14%—supporting continued development [16,24]. Phase II studies (e.g., LUMINOS-101) are ongoing.

Toca 511 (retroviral CD gene therapy). This replication-competent retrovirus delivers cytosine deaminase to tumor cells, converting oral 5-fluorocytosine (Toca FC) into cytotoxic 5-FU locally [31]. In the phase III TOCA-5 trial (n = 403 recurrent high-grade glioma patients post-resection), Toca 511/FC failed to improve OS versus standard therapy (median OS 11.1 vs. 12.2 months) [18], representing a major setback for OV-based prodrug strategies.

Other combinations. Numerous exploratory efforts combine OVs with standard-of-care modalities, targeted agents, or immunotherapies (e.g., DNX-2401 with radiotherapy or small-molecule inhibitors, oHSV with dendritic cell vaccines). Most remain in phase I/II testing.

To date, most OV trials in GBM remain in early development, with few advancing to pivotal phase III evaluation. Landmark successes such as G47Δ (Japanese approval) and T-VEC (melanoma precedent) highlight the translational viability of OVs, but durable OS gains in GBM await validation through larger, rigorously controlled studies.

**Figure 2:**
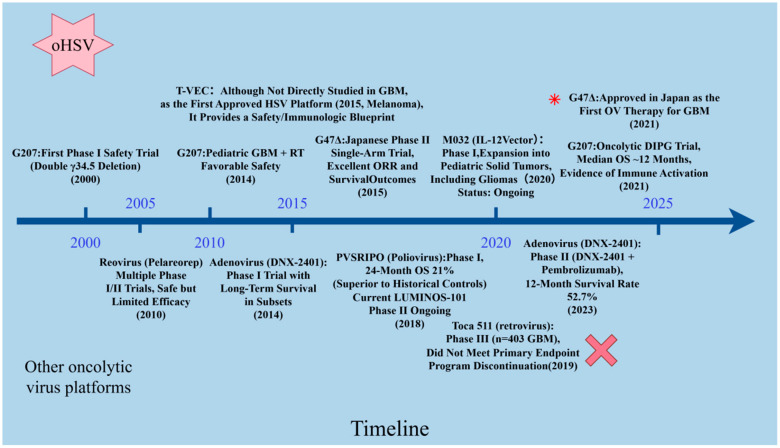
Clinical development timeline of oncolytic viruses (OVs) for glioblastoma. Horizontal arrows (→) indicate the progression of clinical development for each viral platform. Key: ★ indicates regulatory approval or major success; ✗ indicates trial failure or program discontinuation. oHSV vectors (top) have progressed furthest, with G47Δ approved in Japan for recurrent GBM in 2021, representing the first licensed OV for brain tumors. Other viral platforms, including adenovirus (DNX-2401), reovirus (Pelareorep), poliovirus (PVSRIPO), and retroviral constructs (Toca 511), have shown variable outcomes, with ongoing phase II/III trials testing combinatorial strategies.

Figure 2 summarizes representative GBM trials by platform and highlights timing of combination partners.

## 4. Preclinical Study on Mechanisms and Rational Combination Strategies of Oncolytic Virus Platforms in Glioblastoma

Oncolytic virotherapy in glioblastoma (GBM) engages a dynamic interplay between direct oncolysis and immune activation, with immune interactions serving as a central driver of therapeutic efficacy. At the cellular level, OVs replicate within tumor cells, inducing immunogenic cell death that releases tumor-associated antigens (TAAs) and danger-associated molecular patterns (DAMPs). These signals are captured by antigen-presenting cells, such as dendritic cells, which prime T-cell-mediated systemic antitumor immunity, thereby disrupting GBM’s entrenched immune tolerance characterized by local immunosuppression and systemic T-cell sequestration [4,32,33].

OV therapy significantly enhances immune infiltration within the tumor microenvironment, increasing CD4^+^ and CD8^+^ T lymphocytes while suppressing regulatory T cells (Tregs). For instance, biopsies following G47Δ administration revealed substantial rises in CD4^+^/CD8^+^ T-cell densities with persistently low FoxP3^+^ levels, effectively reprogramming the immunosuppressive landscape toward an inflamed phenotype that sensitizes GBM to checkpoint inhibitors [1,4,29,34]. The rational design of OVs increasingly incorporates immunomodulatory payloads to amplify these interactions, with mechanistic underpinnings and combination strategies detailed in Table 3.

At the platform level, each OV vector confers distinct advantages in modulating immune responses. oHSV exhibits broad cellular tropism and potent lytic effects at low multiplicities of infection, while safety switches like acyclovir provide pharmacologic control [3]. Notably, preexisting HSV-1 immunity does not impair efficacy, as systemic antibodies fail to block local viral spread within tumors [3]. The large HSV genome enables the insertion of diverse payloads—such as cytokines, checkpoint inhibitors, or antibodies—making it an ideal armed viral chassis [1]. For example, Xu et al. engineered oHSV to express a full-length anti-CD47 antibody, facilitating sustained intratumoral antibody release, enhanced macrophage and NK cell activity, and prolonged survival in murine GBM models [1].

Despite these strengths, OV therapy faces challenges rooted in GBM’s immunosuppressive microenvironment. Tumors are enriched with M2-polarized macrophages, Tregs, and suppressive cytokines (e.g., TGF-β, IL-10), which establish potent local and systemic immunosuppression that can neutralize viral activity upon systemic administration [4]. Delivery limitations further constrain efficacy; intracranial methods like stereotactic injection offer only regional coverage, while systemic delivery is hindered by the blood–brain barrier and rapid clearance by host defenses [28]. Preclinical studies indicate that intravenously injected viruses are largely sequestered in organs like the liver and spleen, with minimal reach to brain tumors.

Safety and patient stratification represent additional hurdles. HSV-based vectors may incite neuroinflammation, necessitating monitoring and antiviral strategies, though toxicities are typically mild (e.g., fever, leukopenia) [29]. Viral shedding and ectopic transgene expression require careful construct design to mitigate risks. GBM heterogeneity demands biomarker-guided patient selection, as tumors with cold immune profiles or unfavorable molecular signatures may be refractory. Research is exploring biomarkers such as gene expression, immune infiltration indices, and receptor status (e.g., CD155) for stratification.

Mechanisms of failure often relate to pseudoprogression, heterogeneous viral distribution, or premature clearance by host immunity. Most OV trials report disease stabilization rather than complete responses, with long-term survivors in G47Δ studies often having smaller baseline tumor burdens, highlighting volume as an efficacy determinant [29]. Combination strategies with temozolomide or radiotherapy synergize by debulking tumors, inducing immunogenic cell death, and enhancing vascular permeability, collectively facilitating drug penetration and immune activation.

In summary, OVs reshape GBM’s immune landscape through direct oncolysis and multifaceted immune interactions, with platform-specific advantages and combinatory potential outlined in Table 3. Future directions emphasize biomarker-driven personalization and engineered vectors to overcome existing barriers.

## 5. Future Directions

(1) Programmable viral chassis. Rationally engineered “smart” viruses incorporate conditional replication logic, activating only under tumor-specific signals to reduce off-target toxicity. Incorporation of synthetic biology elements (e.g., genetic logic gates, CRISPR-based targeting) may enhance selectivity. Armed constructs are expanding beyond cytokines to encode checkpoint inhibitors, stromal remodelers, or bispecific antibodies. Recent work demonstrates that oHSV encoding anti-CD47 IgG1 functions as a sustained intratumoral antibody factory, potentiating innate and adaptive antitumor immunity [1].
(2) Innovative delivery strategies. To bypass the BBB and enhance biodistribution, approaches include nanoparticle or liposome encapsulation, as well as cellular carriers such as mesenchymal stem cells (MSCs) with tumor-homing properties [28,35]. MSC-based “Trojan horse” vectors may improve systemic delivery. Novel routes—such as intranasal administration or focused ultrasound-mediated BBB disruption—are also under development.
(3) Rational combinations. Evidence suggests OV synergy with immune checkpoint inhibitors, CAR-T cells, radiotherapy, or targeted therapies. For example, DNX-2401 combined with PD-1 blockade has yielded notable survival benefit [23]. OV-mediated antigen release may further enhance CAR-T efficacy, motivating clinical exploration of OV–CAR-T co-therapy [36]. Optimizing sequencing, dosing, and combinatorial regimens will be critical to maximize synergy and overcome GBM’s immune resistance, exemplified by oncolytic adenoviruses encoding bispecific T cell engagers to modulate the tumor microenvironment [37].
(4) Biomarker-driven personalization. Future studies will emphasize biomarker-guided patient stratification. Genomic signatures (e.g., tumor mutational burden, interferon response), receptor expression, and immunohistochemical markers may inform responsiveness to OVs. Circulating biomarkers such as ctDNA or cytokine panels may provide real-time readouts of therapeutic efficacy and prognosis [28].
(5) Expanded indications and regulatory momentum. With recent regulatory milestones (e.g., G47Δ approval in Japan), global development and trial registration of OVs are accelerating. New platforms beyond HSV are under exploration, while lessons from failed programs (e.g., Toca 511 phase III [18]) underscore the need for optimized design and robust trial methodology. Phase III validation remains essential to secure widespread adoption.

## 6. Conclusions

Oncolytic virotherapy represents a transformative paradigm for GBM treatment. Multiple phase I/II trials have demonstrated favorable safety and survival extension in selected patients, with landmark examples such as G47Δ and DNX-2401 providing proof-of-concept. Among platforms, oHSV stands out for its safety profile, genetic flexibility, and therapeutic versatility. Despite persistent challenges—including immune suppression and delivery barriers—rapidly expanding translational and clinical research is actively addressing these limitations [28]. The integration of biomarker-guided stratification, programmable viral engineering, and rational multimodal therapy design promises to elevate OV therapy into a clinically impactful modality. As clinical validation deepens, OVs are poised to become an integral component of GBM management, opening new therapeutic horizons for patients with this intractable malignancy.

## Figures and Tables

**Table 1:** Key characteristics of oncolytic virus platforms investigated for glioblastoma.

Platform (Family; Genome)	Cargo Capacity	Key Modifications
HSV-1 (oHSV; dsDNA)	Large (>30 kb)	γ34.5 and ICP6 deletions; ICP47 deletion
Adenovirus (CRAd; dsDNA)	Moderate (~7.5 kb)	Δ24 E1A deletion; RGD-modified fibers
Reovirus (dsRNA)	Minimal	Native RAS-selective tropism
NDV (−ssRNA)	Minimal	Fusogenic strains
Vaccinia virus (dsDNA)	Very large (>25 kb)	Large genome enabling engineering
PVSRIPO (+ssRNA)	Minimal	Rhinovirus IRES replacement; CD155 targeting
Measles virus (−ssRNA)	Minimal	Edmonston lineage

*Abbreviations*: oHSV, oncolytic herpes simplex virus; dsDNA, double-stranded DNA; CRAd, conditionally replicative adenovirus; dsRNA, double-stranded RNA; NDV, Newcastle disease virus; ssRNA, single-stranded RNA; +ssRNA, positive-sense single-stranded RNA; −ssRNA, negative-sense single-stranded; RNAPVSRIPO, poliovirus sabin-rhinovirus recombinant; RGD, Arg-Gly-Asp; IRES, internal ribosome entry site; CD155, cluster of differentiation 155.

**Table 2:** Representative clinical trials of oncolytic viruses in glioblastoma (GBM): design, delivery, and outcomes.

Platform/Agent	Trial (Phase; N) and Identifier	Setting/Line	Delivery and Dosing	Primary Endpoints	Efficacy Summary	Safety Summary	Notes/Source
**oHSV (teserpaturev, G47Δ)**	Single-arm Phase II; n = 19	Residual or recurrent adult GBM	Stereotactic intratumoral; ≤6 injections	1-yr OS	1-yr OS 84.2%; median OS 20.2 mo; disease control 95%	Mostly grade 1–2 pyrexia, vomiting, leukopenia; manageable	Approved in Japan (2021). Pivotal data in Nature Medicine with identical figures [18]
**oHSV (G207, adults ± RT)**	Phase I; n ≈ 9	Recurrent adult GBM	Intratumoral infusion + single 5 Gy RT boost	Safety, feasibility	Radiographic responses; biological activity observed	No encephalitis; acceptable AE profile	Early “virus + focal RT” experience in adults [19]
**oHSV (G207, pediatrics)**	Phase I; n = 12 (11 evaluable)	Recurrent/progressive pediatric HGG	Intratumoral via catheter; ±5 Gy RT	Safety; biological activity	Median OS 12.2 mo (95% CI 8.0–16.4); 4/11 alive ≥ 18 mo; ↑ TILs	No DLTs; tolerable	NEJM study demonstrating immunologic conversion in pediatric HGG [20]
**oHSV (CAN-3110; nestin-controlled ICP34.5)**	Phase I; n = 41	Recurrent HGG/rGBM	Single stereotactic intratumoral injection; 10^6^–10^10^ PFU	Safety; translational immunology	HSV-1 seropositivity linked to prolonged OS; TCR diversification post-dose	No DLTs; well tolerated	Nature (2023) mechanistic correlative trial; ongoing expansion/fast-track [21]
**oHSV (M032; IL-12-expressing)**	Phase I (ongoing)	Adult HGG	Intratumoral IL-12-armed oHSV	Safety (DLTs); RP2D	Early reports: acceptable safety; results pending	No DLTs to date (interim)	Registered design and early safety reporting. (NCT02062827)
**Adenovirus (DNX-2401)**	Phase I; n = 37 (two cohorts)	Recurrent HGG/GBM	Single intratumoral injection (±resection + reinjection)	Safety; ORR	20% survived >3 yrs after single injection	Favorable profile	Landmark JCO study establishing durable benefit in a subset [22]
**DNX-2401 + Pembrolizumab**	Multicenter Phase II (KEYNOTE-192); n = 49	Recurrent GBM	Intratumoral DNX-2401 → IV pembrolizumab (q3w)	ORR; OS-12	ORR 10.4%; OS-12 52.7%; median OS 12.5 mo; responders lived >3 yrs	Well tolerated; no DLTs	Nature Medicine 2023; prototypical OV + PD-1 synergy signal [23]
**PVSRIPO (modified poliovirus)**	Phase I; n = 61 (NCT01491893)	Recurrent GBM	Convection-enhanced infusion (CED)	MTD/RP2D; OS	OS plateau 21% at 24–36 mo	19% grade ≥ 3 AEs; one hemorrhage at high dose	NEJM 2018 with long-tail survival plateau [24]
**Reovirus (pelareorep)**	Phase Ib (ReoGlio); n = 15	Newly diagnosed GBM (with CRT + TMZ)	GM-CSF (d1–3); IV pelareorep (wks 1 and 4) + CRT + TMZ	Feasibility; PFS (exploratory)	mPFS ≈ 8 mo; dose–response trend	Well tolerated with CRT/TMZ	Peer-reviewed feasibility + sponsor releases; larger trials needed [25]
**NDV (NDV-HUJ)**	Early Phase I/II; n ≈ 11–14	Recurrent GBM/HGG	Repeated IV dosing	Safety; signals of activity	Case responses reported; limited efficacy	Acceptable	Foundational IV NDV experience; human data remain sparse [26]
**Measles virus (MV-CEA)**	Phase I; n = 22	Recurrent GBM	Intracavitary alone vs. intra + cavity dosing	Safety (MTD); OS (exploratory)	mOS 11.6 mo; 1-yr OS 45.5%	No DLTs; predictable AESIs	Nature Communications 2024; modern design with surgical cavity dosing [27]

*Abbreviations:* GBM, glioblastoma; OVs, oncolytic viruses; oHSV, oncolytic herpes simplex virus; PD-1, programmed cell death protein 1; cGAS-STING, cyclic GAMP synthase-stimulator of interferon genes; IFN-I, interferon type I; CTLA-4, cytotoxic T-lymphocyte-associated protein 4; PD-L1, programmed death-ligand 1; MHC-I, major histocompatibility complex class I; Treg, regulatory T cells; MDSC, myeloid-derived suppressor cells; CED, convection-enhanced delivery; RT, radiotherapy; Gy, gray; OS, overall survival; ORR, objective response rate; mOS, median overall survival; mPFS, median progression-free survival; CI, confidence interval; HGG, high-grade glioma; DLT, dose-limiting toxicity; RP2D, recommended phase 2 dose; ↑, increase.

**Table 3:** Mechanisms and rational combination strategies of oncolytic virus (OV) platforms in glioblastoma.

OV Platform	oHSV (G47Δ, CAN-3110, M032, G207)	Adenovirus (DNX-2401)	PVSRIPO (Poliovirus Chimera)	Reovirus (Pelareorep)	NDV (HUJ, Fusogenic Strains)	Vaccinia Virus (Armed)
Core engineering/payloads	γ34.5/ICP6 deletion; ±ICP47 deletion; payloads: GM-CSF, IL-12, antibodies (e.g., anti-CD47)	Δ24 E1A deletion; RGD-modified fibers; payloads: IL-12, CD40L	Rhinovirus IRES replacement; CD155 targeting	Native oncotropism; IV dosing ± GM-CSF	Attenuated/lentogenic strains; fusogenic variants	Large cargo; GM-CSF, IL-2/12/21; bispecifics
Main mechanisms in GBM	Potent oncolysis; DAMP/TAA release; ↑ MHC-I; TME “heating” (↑ CD8, ↓ Treg/MDSC)	Oncolysis; immunogenic cell death; DC priming	Type-I IFN induction; immune reset; bystander killing	Systemic innate activation; RAS-selective replication	Fusogenic oncolysis; type-I IFN activation	Rapid lysis; strong antigen presentation; vascular remodeling
Rational combinations	Anti-PD-1 after priming; peri-OV low-dose RT; OV → CAR-T; ±anti-VEGF	DNX-2401 → anti-PD-1; RT synergy; ±CD40 agonism; TMZ (MGMT-methylated)	PVSRIPO → anti-PD-1 (delayed); RT; STING/TLR agonists	Reovirus → anti-PD-1; CRT/TMZ backbone; ±GM-CSF	NDV → anti-PD-1; RT synergy; ±TLR3 agonists	Vaccinia → anti-PD-1; RT; bispecific-armed vectors
Key limitations/safety	Cerebral edema, PSP; seizure risk; herpetic lesions—acyclovir control	Flu-like AEs; edema; limited parenchymal spread	Procedure-related hemorrhage; neutralizing antibodies	Generally safe; neutralized by pre-existing antibodies; BBB limits	Systemic reactogenicity; neutralization; sparse clinical data	Reactogenicity; neurotropism risk; managed with antivirals
Priority translational readouts	CD8/Treg ratio; PD-L1 induction; TCR clonality expansion; spatial IHC/IMC of DC/TAM; IFN-γ/CXCL9/10; single-cell/Visium signatures; rCBV/rCBF and iRANO concordance	OS-12 vs. historical; intratumoral Ad DNA kinetics; DC activation (CD86), CXCL10; TCR expansion; MRI texture/volumetrics [9]	Long-tail OS plateau; ISG surge; myeloid activation signatures; CSF cytokines; catheter tract coverage maps [35]	Blood viral RNA/DNA; IFN-α/β, IL-6 trajectories; peripheral NK activation; mPFS with CRT [32]	IFN gene signatures; chemokine induction; changes in myeloid subsets [12]	Cytokine panels; endothelial/VECAM changes; intratumoral payload expression; imaging theranostics (if NIS payload) [30]

*Abbreviations*: OV, oncolytic virus; oHSV, oncolytic herpes simplex virus; GBM, glioblastoma multiforme; BBB, blood-brain barrier; HSV, herpes simplex virus; IFN, interferon; IL, interleukin; MDSC, myeloid-derived suppressor cell; Treg, regulatory T cell; PD-1, programmed cell death protein 1; TCR, T-cell receptor; TAA, tumor-associated antigen; VEGF, vascular endothelial growth factor; GM-CSF, granulocyte-macrophage colony-stimulating factor; TAM, tumor-associated macrophage; RT, radiotherapy; CRT, chemoradiotherapy; AE, adverse event; ↑, increase; ↓, decrease.

## References

[1] Xu B., Tian L., Chen J., Wang J., Ma R., Dong W., Li A., Zhang J., Antonio Chiocca E., Kaur B. (2021). An oncolytic virus expressing a full-length antibody enhances antitumor innate immune response to glioblastoma. Nat. Commun..

[2] Yang R., Hedberg J., Montagano J., Seals M., Puri S. (2025). Oncolytic Virus Therapies in Malignant Gliomas: Advances and Clinical Trials. Cancers.

[3] Todo T., Ino Y., Ohtsu H., Shibahara J., Tanaka M. (2022). A phase I/II study of triple-mutated oncolytic herpes virus G47∆ in patients with progressive glioblastoma. Nat. Commun..

[4] Suryawanshi Y.R., Schulze A.J. (2021). Oncolytic Viruses for Malignant Glioma: On the Verge of Success?. Viruses.

[5] Tan R.-B., Yap Y.H.-Y. (2025). Talimogene Laherparepvec (T-VEC): Expanding Horizons in Oncolytic Viral: Therapy Across Multiple Cancer Types. Anti-Cancer Agents Med. Chem..

[6] Ebrahimi S., Makvandi M., Abbasi S., Azadmanesh K., Teimoori A. (2020). Developing oncolytic Herpes simplex virus type 1 through UL39 knockout by CRISPR-Cas9. Iran. J. Basic Med. Sci..

[7] Cooke K., Meisen W.H., Mitchell P., Estrada J., Zhan J., Orf J., Li P., de Zafra C., Qing J., DeVoss J. (2021). Oncolytic virus HSV-1/ICP34. 5-/ICP47-/mFLT3L/mIL12 promotes systemic anti-tumor responses and cooperates with immuno-modulatory agents in multiple mouse syngeneic tumor models. Cancer Res..

[8] Coates K., Nibbs R.J., Fraser A.R. (2025). Going viral: Targeting glioblastoma using oncolytic viruses. Immunother. Adv..

[9] De La Nava D., Ausejo-Mauleon I., Laspidea V., Gonzalez-Huarriz M., Lacalle A., Casares N., Zalacain M., Marrodan L., García-Moure M., Ochoa M.C. (2024). The oncolytic adenovirus Delta-24-RGD in combination with ONC201 induces a potent antitumor response in pediatric high-grade and diffuse midline glioma models. Neuro-Oncology.

[10] van der Meulen-Muileman I.H., Amado-Azevedo J., Lamfers M.L., Kleijn A., Idema S., Noske D.P., Dirven C.M., van Beusechem V.W. (2025). Adenovirus-Neutralizing and Infection-Promoting Activities Measured in Serum of Human Brain Cancer Patients Treated with Oncolytic Adenovirus Ad5-∆ 24. RGD. Int. J. Mol. Sci..

[11] Yang A., Wang X., Jin L., Luo H., Yang Z., Yang N., Lin X., Yang Y., Zhao X., He Z. (2024). Human umbilical cord mesenchymal stem cell exosomes deliver potent oncolytic reovirus to acute myeloid leukemia cells. Virology.

[12] Ginting T.E., Christian S., Larasati Y.O., Suryatenggara J., Suriapranata I.M., Mathew G. (2019). Antiviral interferons induced by Newcastle disease virus (NDV) drive a tumor-selective apoptosis. Sci. Rep..

[13] Cuoco J.A., Rogers C.M., Mittal S. (2021). The oncolytic Newcastle disease virus as an effective immunotherapeutic strategy against glioblastoma. Neurosurg. Focus.

[14] Kousar K., Naseer F., Abduh M.S., Anjum S., Ahmad T. (2023). CD44 targeted delivery of oncolytic Newcastle disease virus encapsulated in thiolated chitosan for sustained release in cervical cancer: A targeted immunotherapy approach. Front. Immunol..

[15] Lee N., Jeon Y.-H., Yoo J., Shin S.-k., Lee S., Park M.-J., Jung B.-J., Hong Y.-K., Lee D.-S., Oh K. (2023). Generation of novel oncolytic vaccinia virus with improved intravenous efficacy through protection against complement-mediated lysis and evasion of neutralization by vaccinia virus-specific antibodies. J. Immunother. Cancer.

[16] Wang X., Lu J., Guo G., Yu J. (2021). Immunotherapy for recurrent glioblastoma: Practical insights and challenging prospects. Cell Death Dis..

[17] Anker S.C., Szczeponik M.G., Dessila J., Dittus K., Engeland C.E., Jäger D., Ungerechts G., Leber M.F. (2023). Oncolytic measles virus encoding microRNA for targeted RNA interference. Viruses.

[18] Cloughesy T.F., Petrecca K., Walbert T., Butowski N., Salacz M., Perry J., Damek D., Bota D., Bettegowda C., Zhu J.J. (2020). Effect of Vocimagene Amiretrorepvec in Combination with Flucytosine vs Standard of Care on Survival Following Tumor Resection in Patients with Recurrent High-Grade Glioma: A Randomized Clinical Trial. JAMA Oncol..

[19] Friedman G.K., Bernstock J.D., Chen D., Nan L., Moore B.P., Kelly V.M., Youngblood S.L., Langford C.P., Han X., Ring E.K. (2018). Enhanced Sensitivity of Patient-Derived Pediatric High-Grade Brain Tumor Xenografts to Oncolytic HSV-1 Virotherapy Correlates with Nectin-1 Expression. Sci. Rep..

[20] Friedman G.K., Johnston J.M., Bag A.K., Bernstock J.D., Li R., Aban I., Kachurak K., Nan L., Kang K.D., Totsch S. (2021). Oncolytic HSV-1 G207 Immunovirotherapy for Pediatric High-Grade Gliomas. N. Engl. J. Med..

[21] Ling A.L., Solomon I.H., Landivar A.M., Nakashima H., Woods J.K., Santos A., Masud N., Fell G., Mo X., Yilmaz A.S. (2023). Clinical trial links oncolytic immunoactivation to survival in glioblastoma. Nature.

[22] Lang F.F., Conrad C., Gomez-Manzano C., Yung W.K.A., Sawaya R., Weinberg J.S., Prabhu S.S., Rao G., Fuller G.N., Aldape K.D. (2018). Phase I Study of DNX-2401 (Delta-24-RGD) Oncolytic Adenovirus: Replication and Immunotherapeutic Effects in Recurrent Malignant Glioma. J. Clin. Oncol..

[23] Nassiri F., Patil V., Yefet L.S., Singh O., Liu J., Dang R.M.A., Yamaguchi T.N., Daras M., Cloughesy T.F., Colman H. (2023). Oncolytic DNX-2401 virotherapy plus pembrolizumab in recurrent glioblastoma: A phase 1/2 trial. Nat. Med..

[24] Desjardins A., Gromeier M., Herndon J.E., Beaubier N., Bolognesi D.P., Friedman A.H., Friedman H.S., McSherry F., Muscat A.M., Nair S. (2018). Recurrent Glioblastoma Treated with Recombinant Poliovirus. N. Engl. J. Med..

[25] Kendall J., Chalmers A., McBain C., Melcher A., Samson A., Phillip R., Brown S., Short S. (2020). Ctim-14. Pelareorep and Granulocyte-Macrophage Colony-Stimulating Factor (Gm-Csf) with Standard Chemoradiotherapy/Adjuvant Temozolomide for Glioblastoma Multiforme (Gbm) Patients: Reoglio Phase I Trial Results. Neuro-Oncology.

[26] Abdullah J.M., Mustafa Z., Ideris A. (2014). Newcastle disease virus interaction in targeted therapy against proliferation and invasion pathways of glioblastoma multiforme. Biomed. Res. Int..

[27] Galanis E., Dooley K.E., Keith Anderson S., Kurokawa C.B., Carrero X.W., Uhm J.H., Federspiel M.J., Leontovich A.A., Aderca I., Viker K.B. (2024). Carcinoembryonic antigen-expressing oncolytic measles virus derivative in recurrent glioblastoma: A phase 1 trial. Nat. Commun..

[28] Alomari O., Eyvazova H., Güney B., Al Juhmani R., Odabasi H., Al-Rawabdeh L., Mokresh M.E., Erginoglu U., Keles A., Baskaya M.K. (2025). Oncolytic Therapies for Glioblastoma: Advances, Challenges, and Future Perspectives. Cancers.

[29] Todo T., Ito H., Ino Y., Ohtsu H., Ota Y., Shibahara J., Tanaka M. (2022). Intratumoral oncolytic herpes virus G47∆ for residual or recurrent glioblastoma: A phase 2 trial. Nat. Med..

[30] Nakao S., Arai Y., Tasaki M., Yamashita M., Murakami R., Kawase T., Amino N., Nakatake M., Kurosaki H., Mori M. (2020). Intratumoral expression of IL-7 and IL-12 using an oncolytic virus increases systemic sensitivity to immune checkpoint blockade. Sci. Transl. Med..

[31] Sonoda-Fukuda E., Takeuchi Y., Ogawa N., Noguchi S., Takarada T., Kasahara N., Kubo S. (2024). Targeted Suicide Gene Therapy with Retroviral Replicating Vectors for Experimental Canine Cancers. Int. J. Mol. Sci..

[32] Maddox J.F., Amuzie C.J., Li M., Newport S.W., Sparkenbaugh E., Cuff C.F., Pestka J.J., Cantor G.H., Roth R.A., Ganey P.E. (2009). Bacterial-and viral-induced inflammation increases sensitivity to acetaminophen hepatotoxicity. J. Toxicol. Environ. Health Part A.

[33] White K., Connor K., Meylan M., Bougoüin A., Salvucci M., Bielle F., O’farrell A., Sweeney K., Weng L., Bergers G. (2023). Identification, validation and biological characterisation of novel glioblastoma tumour microenvironment subtypes: Implications for precision immunotherapy. Ann. Oncol..

[34] Kardani K., Sanchez Gil J., Rabkin S.D. (2023). Oncolytic herpes simplex viruses for the treatment of glioma and targeting glioblastoma stem-like cells. Front. Cell Infect. Microbiol..

[35] Zawit M., Swami U., Awada H., Arnouk J., Milhem M., Zakharia Y. (2021). Current status of intralesional agents in treatment of malignant melanoma. Ann. Transl. Med..

[36] Chalise L., Kato A., Ohno M., Maeda S., Yamamichi A., Kuramitsu S., Shiina S., Takahashi H., Ozone S., Yamaguchi J. (2022). Efficacy of cancer-specific anti-podoplanin CAR-T cells and oncolytic herpes virus G47Δ combination therapy against glioblastoma. Mol. Ther. Oncolytics.

[37] Choi M.J., So E.Y., Akosman B., Lee Y.E., Raufi A.G., Bertone P., Reginato A.M., Chen C.C., Lawler S.E., Wong E.T. (2025). Enabling CAR-T Cell Immunotherapy in Glioblastoma by Modifying Tumor Microenvironment via Oncolytic Adenovirus Encoding Bispecific T Cell Engager. bioRxiv.

